# Supplementary figures and images for: Addressing biodiversity knowledge shortfalls in New World Helicopsychidae (Insecta, Trichoptera): Potential distribution, environmental gradients, and identification of conservation and research priority areas
Source: PLoS One. 2025 Jul 28;20(7):e0327580. doi: 10.1371/journal.pone.0327580 (PMC12303351; doi:10.1371/journal.pone.0327580)

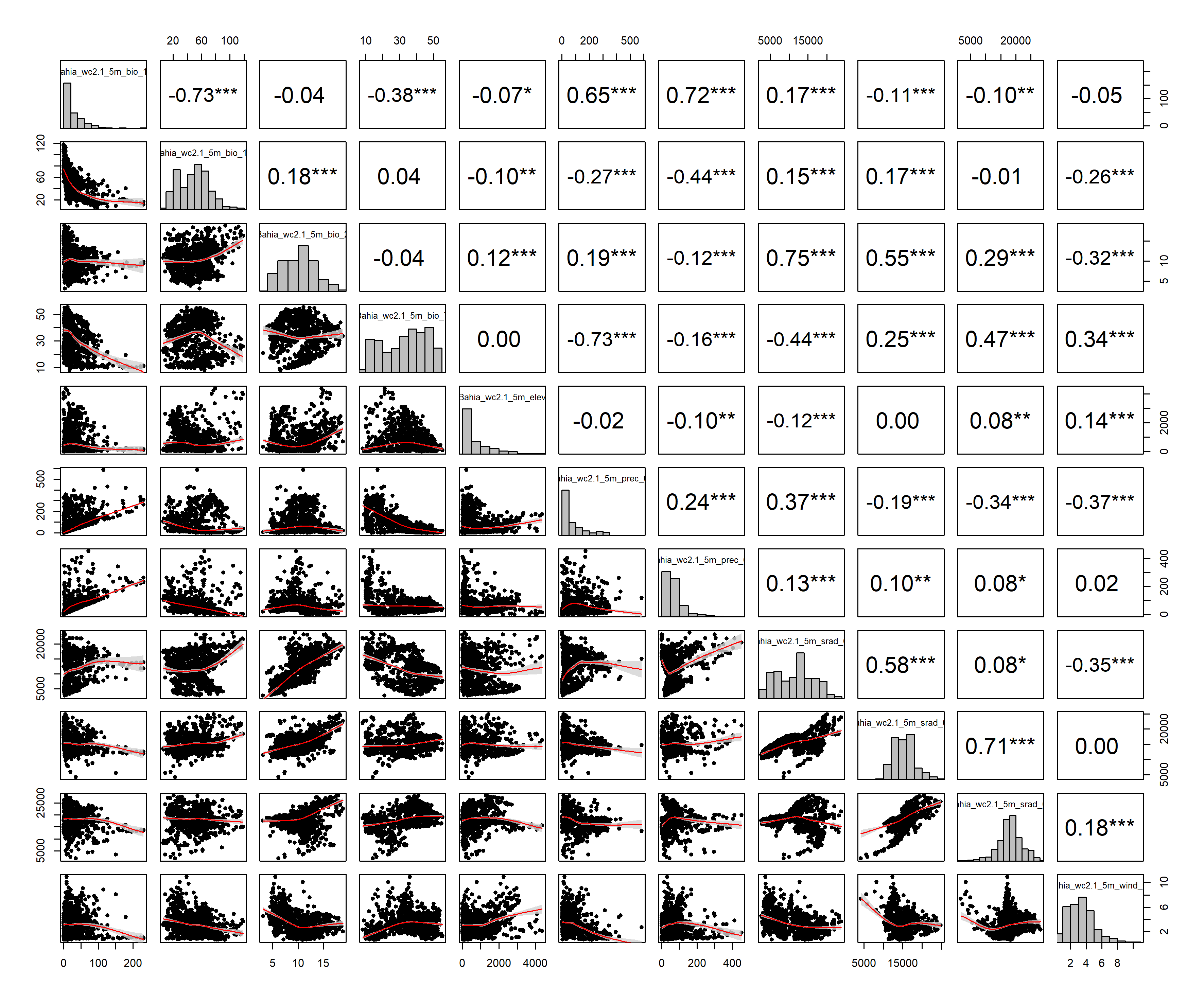

Supplement: S1 Fig — (TIFF) [file pone.0327580.s004.tiff]
