# Supplementary material for: Addressing biodiversity knowledge shortfalls in New World Helicopsychidae (Insecta, Trichoptera): Potential distribution, environmental gradients, and identification of conservation and research priority areas
Source: PLoS One. 2025 Jul 28;20(7):e0327580. doi: 10.1371/journal.pone.0327580 (PMC12303351; doi:10.1371/journal.pone.0327580)
Supplement: Attachment — Submitted filename: Manuscript File_revised.docx [file pone.0327580.s006.docx]

**Addressing biodiversity knowledge shortfalls in New World Helicopsychidae (Insecta, Trichoptera): potential distribution, environmental gradients, and identification of conservation and research priority areas**

**Overview of New World Helicopsychidae: Potential distribution, environmental gradients, and priority areas**

**Rafael Pereira^1,2^* & Adolfo Calor^2^**

^1^Laboratório de Organismos Aquáticos – LOA, Programa de Pós-Graduação em Sistemas Aquáticos Tropicais, Universidade Estadual de Santa Cruz – (PPGSAT – UESC); Campus Prof. Soane Nazaré de Andrade, Km 16 – Rodovia Jorge Amado, Sala 01EA – 1º andar Pav. Max de Menezes CEP 45662-900, Ilhéus – BA, Brazil

^2^Laboratório de Entomologia Aquática – LEAq, Instituto de Biologia, Programa de Pós-Graduação em Biodiversidade e Evolução, Universidade Federal da Bahia (PPGBioEvo, UFBA), Rua Barão de Jeremoabo, 143, CEP 40130-115, Campus Ondina, Salvador – BA, Brazil

*****rafaelpsilvape@gmail.com

Rafael Pereira: <https://orcid.org/0000-0001-7578-6042>

Adolfo R. Calor: <https://orcid.org/0000-0003-3975-3176>

**Abstract**

This study provides an integrative analysis of Helicopsychidae biodiversity in the New World (NW), examining their distribution patterns, environmental gradients, biodiversity hotspots, and biodiversity knowledge shortfalls. We estimated species richness, delineated potential distribution, and identified areas of priority for research/conservation efforts. Our estimates indicate that up to 75% of NW Helicopsychidae species remain undescribed, with a notable Linnean shortfall at the subgenus level: approximately 50% of *H*. (*Cochliopsyche*) species and up to 77% of *H*. (*Feropsyche*) species are yet to be described. Knowledge of semaphoronts is also limited, with immature stages documented for only 15% of *H*. (*Feropsyche*) species and 6% of *H*. (*Cochliopsyche*). Distributional records are concentrated in the Brazilian subregion (between 0°–24°S), and most species have a short environmental distribution gradient. Based on currently known, priority conservation areas are in the Antilles and the Tropical Forest. For *H*. (*Feropsyche*), priority areas include low-altitude and coastal regions with low-order streams, whereas *H*. (*Cochliopsyche*) conservation priorities lie in the large river basins, especially in the Amazon and Atlantic Forests. Future research efforts should focus on *H*. (*Feropsyche*) in the Chaco and high-altitude areas of the Atlantic Forest, Cerrado, and Caatinga domains, as well as on *H*. (*Cochliopsyche*) across South America’s high order river in South American (e.g., Amazon, Orinoco, Paraná, and São Francisco Rivers). Here, highlights recent advances in the taxonomy and distribution cataloguing of NW Helicopsychidae, despite significant progress, up to 75% of Helicopsyche species remain undescribed, emphasizing the need for continued research. Although well-sampled regions like the Antilles, South Central America, Amazon coastal areas, and Central Atlantic Forest Ecological Corridor should be prioritized for conservation, vast areas such as the Amazon lowlands, northeast and southwest Atlantic Forest, and transition areas of Atlantic Forest and Dry Diagonal remain underexplored. To the best of our knowledge, this study represents the first empirical analysis of environmental gradients for Trichoptera species. It establishes a foundation for understanding biogeographic patterns, environmental gradients, and the identification of biodiversity hotspots and potential distribution areas for Helicopsychidae in the New World. This work will guide future research and conservation efforts for Helicopsychidae and other Trichoptera groups in the region.

**Key words**. distribution modelling, Haeckelian shortfalls, Hutchinsonian shortfalls, Linnean shortfalls, snail-case caddisflies, Wallacean shortfalls.

# Introduction

The distributional patterns generally used the distribution of known species as a proxy (Droissart et al. 2018), and, consequently, they are strongly related to biased collection efforts (Kreft & Jetz 2010). There are huge gaps in biodiversity knowledge related to areas neglected in terms of collection effort, fauna inventories, taxonomists, and investment in research, especially in developing countries (Oliveira et al. 2016; Ely et al. 2017). These factors cause collection bias and directly affect biodiversity knowledge (Kier et al. 2005; Collen et al. 2008; Yang et al. 2013).

In this context, some authors have defined the different knowledge gaps, or the Biodiversity Knowledge Shortfalls (BKS), in various categories such as gap of species knowledge is the Linnaean shortfall, the unknown species geographic distribution is the Wallacean shortfall, the gap of knowledge about semaphoronts of species Haeckelian shortfall, the gap of knowledge about abiotic tolerance of species Hutchinsonian shortfall and unknown of evolution of groups is the Darwinian shortfall (Hortal et al. 2015; Faria et al. 2021). Among the tools available to face the biodiversity knowledge shortfalls, distribution modelling can identify areas with a high probability of potential distribution (environmental suitability) (Tessarolo et al. 2021). The generated models can help to prioritize resources and efforts in areas with the probability of potential distribution of species (Terribile et al. 2018).

On the other hand, anthropogenic actions have caused planetary-scale changes, which have caused the sixth mass extinction (Ceballos et al. 2017) with critical loss of biodiversity faster than our ability to catalogue it (Montoya et al. 2018). Among the ecosystems, freshwater environments cover only 1% of Earth’s land surface but comprise around 10% of all species (Strayer & Dudgeon 2010). Levels of endemism in these environments are remarkably high, and about 20% of the New World (NW) species are threatened *sensu* IUCN (Watson et al. 2018). Due to these characteristics, freshwater environments have the most acute biodiversity crisis among ecosystems (Tickner et al. 2020). The formulation of strategies based on large datasets is urgent to protect species, as well as the priority areas (Harper et al. 2021), and consequently reduce (or reverse) the decline of freshwater biota as much as possible (Tickner et al. 2020).

Several insect orders have species with life stages occurring in freshwater ecosystems, but some orders stand out as having primarily aquatic origins (e.g., Trichoptera) (Morse 2017). Among these amphibiotic orders, the caddisflies stand out as having the greatest richness and diversity of functional trophic groups (Morse et al. 2019). Despite recent advances in caddisfly taxonomy and phylogeny, the order is underexplored and poorly known in several aspects (de Moor & Ivanov 2008; Morse 2017; Morse et al. 2019; Thomas et al. 2020, 2023). Knowledge about Trichoptera species, distribution ranges, and biogeographic patterns is scarce, especially in the Neotropical region (NT) (de Moor & Ivanov 2008; Ríos-Touma et al. 2017; Santos et al. 2020).

Studies of Trichoptera have focused almost entirely on systematics, with few studies related to biogeography and a much smaller proportion concerned with understanding which factors limit the distribution of groups (Ríos-Touma et al. 2022). However, this data is essential for understanding how species will behave both in scenarios of environmental impacts (e.g., deforestation, burning) and in scenarios of climate change (Harvey et al. 2023). Especially when it comes to taxa that play a key role in the trophic dynamics of freshwater and adjacent terrestrial ecosystems, such as the Trichoptera (Morse et al. 2019), and with factors such as temperature, rainfall, and riparian vegetation directly influencing the distribution of species (Kusch 2014). Despite its importance, it has suffered alarming population declines, 2/3 (68%) of the caddisfly species experiencing population declines, 63% of the species threatened, and a local/regional extinction rate of 6.8% (Sáchez-Bayo & Wyckhuys 2019).

Among the caddisflies, Helicopsychidae von Siebold, 1856 are represented by two genera, the monotypic *Rakiura* McFarlane, 1973 (endemic to New Zealand), and *Helicopsyche* von Siebold, 1856 (circum-global distribution) (Johanson 1998). *Helicopsyche* comprises 298 valid species in six subgenera: *Cochliopsyche* Müller, 1885, *Feropsyche* Johanson, 1998, *Galeopsyche* Johanson, 1998, *Helicopsyche*, *Petrotrichia* Ulmer, 1910, *Saetotricha* Brauer, 1865 (Morse 2024). In the NW, we have 146 valid species in two subgenera, *Cochliopsyche*, with 17 Neotropical species, and *Feropsyche*, with 129 species (including three fossil species from the Dominican Republic) widely distributed throughout the NW (Nearctic region (NA) with 13 and NT with 125 valid species) (Morse 2024; Pereira & Calor 2024).

*Helicopsyche* is a genus recognized by larval cases built with sand grains, helically organized, and resembling snail shells (Holzenthal & Calor 2017). Like most caddisflies, the immature stages live in freshwater ecosystems and, after completing the metamorphosis, they emerge as winged adults associated with riparian forests (Holzenthal et al. 2015). Due to its wide distribution and occurrence in different freshwater environments of the NW (Johanson 1997), the genus represents a good biological model for initial studies that seek to identify and define distributional patterns of amphibiotic insect groups. Furthermore, understanding how these species are distributed and grouped can provide subsidies for the identification of under-sampled areas (Citation??).

The subgenus *Feropsyche* was revised by Johanson (2002), and subsequent studies have primarily focused on species descriptions and new distribution records (e.g., Dumas & Nessimian 2019; Gama-Neto et al. 2019; Cavalcante-Silva et al. 2022; Moreno et al. 2023; Pereira & Calor 2024). In contrast, *Cochliopsyche* has received comparatively less attention following its revision by Johanson (2003), with most contributions limited to new distribution records (e.g., Souza et al. 2013; Desidério et al. 2017; Razo-Gonzáles et al. 2023) and the description of a single new species, *Helicopsyche* (*Cochliopsyche*) *nyurga* Oláh & Oláh, 2022. The taxonomic literature on *Helicopsyche* also reveals a notable bias toward male specimens (Johanson 2002; Holzenthal & Calor 2017).

Species description based only on male adults is a quite frequent practice in caddisfly taxonomy and accurately separates species (Santos et al. 2020), as in some other insect orders (Yang et al. 2013). For the most caddisflies, immature stages and females are unknown (an average of <15% of the immatures are known, and in 41 Neotropical genera, the immature stages are unknown) (Pes et al. 2018), demonstrating a knowledge gap related to other semaphoronts. This causes a genuine problem since most adult collections use light traps, which collect more adult females than males (Larsson et al. 2019). In addition, *Helicopsyche* species are mainly reported exclusively to type localities and/or adjacent localities (like the same district) (e.g., Pereira & Calor 2024). The combination of these two factors inevitably leads to knowledge shortfalls (Hortal et al. 2015).

Understanding distribution patterns, environmental gradients, and biodiversity hotspots—along with identifying Biodiversity Knowledge Shortfalls (KBS) and prioritizing research efforts—provides crucial insights for addressing Wallacean and Hutchinsonian shortfalls and informs strategies for tackling other BKS. Additionally, this information forms a foundation for developing new research proposals, conservation actions, and enhancing understanding of biogeographic patterns and the relationships between species and regions for New World Trichoptera. Within this framework, this study aimed to identify distribution patterns, establish environmental gradients, and model the distribution of Helicopsychidae subgenera in the New World.

# Materials and methods

*Study area*

The New World is divided into two regions: the Nearctic and Neotropical regions, with a transition zone located in the western and eastern Sierra Madre of Mexico (Morrone 2014). This zone serves as a pathway connecting elements of the Nearctic and Neotropical fauna (Morrone 2014). Following these delimitations and using the distribution of Trichoptera as a proxy de Moor & Ivanov (2008) divided NA into three sub-regions: (i) Nearctic East (=Californian+Rocky Mountain), (ii) Nearctic West (=Alleghany), and (iii) Beringian (=Canadian) (14). As for the NT caddisflies, the fauna is divided into two subregions: Brazilian (= Brazilian + Mexican + Antillean) and Patagonian (= Chilean). Apart from these, all other proposals for bioregionalization for aquatic insects have been made with too large divisions (e.g., Vinson & Hawkins 2003), making it difficult to use. Here we'll follow the gradients of de Moor & Ivanov’ proposal.

*Distributional data and richness estimates*

The distribution data basis was compiled through the primary literature (description and record of the occurrence of species), the database of Global Biodiversity Information Facility (GBIF, https://www.gbif.org), and SpeciesLink (<http://www.splink.org.br/>). Original data from collections at the Museu de História Natural da Bahia, Universidade Federal da Bahia (UFBA), and the Laboratório Citotaxonomia e Insetos Aquáticos do Instituto Nacional de Pesquisa da Amazônia (LACIA-INPA). The data used in this study comes from databases and material deposited in museums and collections, and no licenses are required.

We use gazetteers and Google Maps© to register localization without coordinates; the centroid of the less comprehensive location was used. After the data compilation, two-stage filtering process was performed, (i) manual selection of the data, discarding points without coordinate information, generic data of locality (e.g., only state), or with the indeterminacy of the species (e.g., identification only to the genus level), and (ii) selection in R environment, discarding points that can generate an analysis bias (e.g., points localized at the centroid of the capital areas and/or with same coordinates or in marine areas). Species distribution map and heatmap (Kernel density) were prepared using QGIS v. 3.10.10.

For estimating the number of unknown species of *Feropsyche* in NW and *Cochliopsyche* in NT, the distributional records and bioregions delimited here are used, using non-parametric estimators. Estimators were calculated based on incidence data (presence-absence only), using freshwater ecoregions (Abell et al. 2008) as sampling unities, counting the occurrences in each one, with the function ‘specpool' from the ‘vegan’ package (Oksanen et al. 2019) in the R environment. This function calculates three estimators of species richness: CHAO2, second-order jackknife (JACK2) (more adequate for incidence data) (Kindt & Coe 2005). These non-parametric estimators are useful to estimate a potential number of unobserved species based on incidence data as those available here, and they have shown better performance than model-based or asymptotic estimators (Palmer 1990, Hortal et al. 2006).

*Environmental gradients*

To obtain the standards of distribution and distribution range regarding temperature, precipitation, elevation, and characteristics of freshwater environments, a dataset was produced consisting of distribution data, biogeoclimatic data (i.e., temperature, precipitation, elevation) obtained through WorldClim version 2.1 (https://www.worldclim.org/data/worldclim21.html), and data of freshwater ecosystems (i.e., Sthaler order, flow velocity, discharge, water body size) available in HydroRIVERS and HydroLAKES version 1.0 (https://www.hydrosheds.org). Based on this, distribution value plots and boxplots were generated using the 'ggplot' function of the ggplot2 package, a correlation analysis between the variables was performed with the Spearman method using the 'correlate' function of the 'corrr' package (https://cran.r-project.org/package=corrr) and PERMANOVA analysis with Jaccard similarity index was performed to assess the similarity between subgenus both performed in R environment.

*Distribution Modelling*

Environmental data were obtained from monthly climate data for minimum, mean, and maximum temperature, precipitation, solar radiation, wind speed, water vapor pressure, and for total precipitation, 19 “bioclimatic” variables, and elevation on a scale of 5 arc minutes, all environmental variables obtained in WorldClim version 2.1 (https://www.worldclim.org/data/worldclim21.html). The resolutions of these environmental variables were kept, avoiding the loss of information or the impossibility of modelling. After obtaining the data, a correlation analysis between the variables was performed with the Spearman method using the 'correlate' function of the 'corrr' package (https://cran.r-project.org/package=corrr) in the R environment. This correlation analysis permitted to selection of uncorrelated variables and consequently to avoid overweighing in the models. Variables with correlation values greater than 80% were considered correlated.

Four correlative modelling algorithms were used: Bioclim (Nix 1986), Domain (Carpenter et al. 1993), Generalized Linear Model (GLM) (Nelder 1972), and the Vector Support Machine (SVM) (Tax & Duin 2004). As absence data is not predicted or lacks accuracy given the bias/ scarce sampling scenario, presence/pseudo-absence models will be used. We randomly generated pseudo-absence points (1:1 ratio for the occurrence points) (VanDerWal et al. 2009), through the 'randomPoints' function of the 'dismo' package (https://cran.r-project.org/package=dismo) in the R environment. Data partitioning was randomly performed in 70% for training and 30% for testing the models. The repeatability of the models (200 times) was used to increase the robustness of the result. After the models were evaluated using the Area Under the Curve (AUC) (Hanley & McNeil 1982) method, which only models with values of AUC >80% were used for the construction of suitability maps. Finally, we used the default limits of presence and absence for the construction of the suitability maps.

# Results

*Biodiversity knowledge shortfalls*

In the New World, the *Helicopsyche* species richness remains largely underestimated, with estimates suggesting up to 571 species (error = 142), indicating that approximately 75% of the species remain undescribed (Table 2).

The subgenus *Cochliopsyche* comprises 17 valid species, all distributed in the Neotropics, particularly in the Brazilian subregion (Fig. 1B). Estimates project up to 36 species (error = 16), with 53% remaining undescribed (Table 2). The first species was described by Ulmer in 1905, followed by significant contributions from Flint (1972, 1983, 1986). Johanson's revision added 12 species, and after a 20-year gap, Oláh & Oláh (2022) described a new species from Ecuador (Fig. 2C).

The subgenus *Feropsyche* includes 129 valid species, widely distributed across tropical regions of the Nearctic and Neotropical zones, with the highest richness in the Brazilian subregion (127 species) (Fig. 1G). Estimates suggest up to 553 species (error = 152), with 77% remaining undescribed (Table 2). In the past 20 years, about 40% of *Feropsyche* species were described, averaging five species annually, compared to two species per year from 1840 to 1999 (Fig. 2D).

Approximately 79% of *Helicopsyche* species have restricted distributional records (< 10 records), with around 70% occurring only in type localities or adjacent areas, such as the same stream (Figs 2A and B; Table S1). The species with the widest distribution are *H*. (*Cochliopsyche*) *opalescens* Flint, 1972, with 41 records across 147.3 km², and *H*. (*Feropsyche*) *borealis* (Hagen, 1861), with 48 records across 211.03 km² (Figs 2A, 2B). In *Cochliopsyche*, 58% of species have restricted records, with 47% limited to type localities or adjacent areas (Fig. 2A; Table S1). In contrast, *Feropsyche* shows a higher percentage of species with restricted records, reaching 88%, and 72% of these species are confined to type localities or adjacent areas (Fig. 2B; Table S1).

Knowledge of semaphoronts remains limited, with larval, pupal, and female stages unknown for most species (Holzenthal & Calor, 2017; Calor et al., 2024; Morse, 2024). Immature stages are documented for 20 valid species (15%), and female adults are described for 30 valid species (24%). In *Cochliopsyche*, adult males of all species are described. However, immature stages are known for only one species, *H*. (*Cochliopsyche*) *vazquezae* (Flint, 1986), and females are documented for two species, *H*. (*Cochliopsyche*) *clara* (Ulmer, 1905) and *H*. (*Cochliopsyche*) *vazquezae* (Table 1). In *Feropsyche*, males of 121 species and females of 28 species are described, including two species known solely from females: *H*. (*Feropsyche*) *lutea* (Hagen, 1861) and *H*. (*Feropsyche*) *minuscula* Martynov, 1912. Immature stages have been documented for 19 species of *H*. (*Feropsyche*). Of these, seven species are known exclusively from their larval cases: *H*. (*Feropsyche*) *braziliensis* (Swainson, 1840), *H*. (*Feropsyche*) *colombiensis* von Siebold, 1956, *H*. (*Feropsyche*) *helicoidella* (Vallot, 1855), *H*. (*Feropsyche*) *minima* von Siebold, 1956, *H*. (*Feropsyche*) *scalaris* Hagen, 1864, *H*. (*Feropsyche*) *thelidomus* Hagen, 1864, and *H*. (*Feropsyche*) *umbonata* Hagen, 1864 (Table 1).

Regarding environmental gradients, *Helicopsyche* species occur along an elevation gradient from 0 to 5042 m a.s.l. *Cochliopsyche* species range from 3 to 2457 m, with 80% found below 600 m (Fig. 3A). In contrast, *Feropsyche* species range from 0 to 5042 m, with 45% of records above 600 m (Fig. 3B), showing significant differences between subgenera distributions (F(1)=35.19, p-value=0.001).

*Helicopsyche* species are found across the 1st to 10th Strahler stream orders. *Cochliopsyche* has nearly half of its records above 4th order (45%), with a range from 1st to 10th order (Fig. 3C). Conversely, *Feropsyche* has less than 20% of its records above 4th order, with a range from 1st to 8th order (Fig. 3D), indicating significant differences (F(1)=45.93, p-value=0.001).

The species occur in areas with annual precipitation ranging from 349 to 4427 mm³/year. *Feropsyche* has 47% of its records in areas with less than 1000 mm³/year (range 349–4427 mm³/year) (Fig. 4A), while *Cochliopsyche* has 74% of its records in areas above 1000 mm³/year (range 959–3798 mm³/year) (Fig. 4B), with significant differences (F(1)=58.52, p-value=0.001).

Temperature gradients from -17°C to 40°C. *Feropsyche* is the only subgenus found in areas with negative temperatures, with a range from -17°C to 40°C (Fig. 4C). In contrast, *Cochliopsyche* species are found between 5°C and 35°C (Fig. 4D), with significant differences (F(1)=55.10, p-value=0.001).

*Distribution patterns of New World*

Distributional records of 146 species (17 *Cochliopsyche* and 129 *Feropsyche*) and six undescribed species (five *Cochliopsyche* and one *Feropsyche*) were catalogued (Table S1). The northernmost species of *H*. (*Feropsyche*) is *H*. (*Feropsyche*) *borealis*, recorded in Canada at approximately 47°N, while the southernmost is *H*. (*Feropsyche*) *caligata*, found in Chile at around 39°S. In *H*. (*Cochliopsyche*), the northernmost species is *H*. (*Cochliopsyche*) *vazquezae* from Mexico (17°N), and the southernmost is *H*. (*Cochliopsyche*) *opalescens* from Uruguay (32°S). The highest species richness in both subgenera is concentrated between latitudes 0° and 24°S (Fig. 1).

In the New World, biodiversity hotspots *H*. (*Feropsyche*) are concentrated in three main regions: (i) northwestern Colombia extending through Costa Rica, Nicaragua, and Panama, with 231 records representing 25 species; (ii) the Greater Antilles, with 165 records of 22 species; and (iii) eastern Brazil, with 93 records of 13 species (Fig. 2A). For *H*. (*Cochliopsyche*), three prominent hotspots include: (i) northern Peru, much of Colombia, and Ecuador, with some extension into the Amazon basin; (ii) the Central Amazon in Brazil; and (iii) eastern Brazil, encompassing the Central Atlantic Forest and high-altitude areas of the Caatinga domain (Fig. 2B).

Species of *Helicopsyche* occur across 90 freshwater ecoregions (sensu Abell et al. 2008). The Northeastern Mata Atlântica Freshwater Ecoregion harbors the highest species richness (23 spp.), followed by the South America Caribbean Freshwater Ecoregion (15 spp.), and both the Amazonas Lowlands and Paraíba do Sul Freshwater Ecoregions (14 species each). *Helicopsyche* (*Cochliopsyche*) is predominantly recorded from the Amazonas Lowlands (10 spp.) and Northeastern Mata Atlântica (8 spp.), whereas *H*. (*Feropsyche*) is more frequent in the South America Caribbean (15 spp.), San Juan (Nicaragua and Costa Rica), and Paraíba do Sul Freshwater (12 species each) ecoregions (see Table S2 for details).

*Distribution modelling*

After conducting correlation testing, nine raster variables were found to be uncorrelated, belonging to five groups (bioclimatic, elevation, precipitation, solar radiation, and wind speed), as listed in Table 3 (for more details, see S3 Table and S4 Fig.). Among the four algorithms tested, all yielded AUC values higher than the cut-off value (S5 Fig.). These values were subsequently used to generate environmental suitability maps for the subgenera (Fig. 1A, D).

Distributional records of 129 species (112 *Feropsyche* and 17 *Cochliopsyche*), along with six undescribed species (one *Feropsyche* and five *Cochliopsyche*). The distribution modelling indicates higher environmental suitability (>0.7) for *Feropsyche* species in NA areas: (i) South Florida (Florida Keys) (lacking species records), while in the Neotropical region, four areas also exhibit high environmental suitability: (ii) nearly the entire continental Central America, (iii) the Greater and Lesser Antilles, coastal regions of (iv) northwestern South America, (v) east of the Andes in the Chaco domain, and (vi) almost all areas within the Atlantic Forest domain, high-elevation areas to the east of the Cerrado and Caatinga domains (Fig. 1A, D). For *Feropsyche* species, low suitability is observed in three areas: (i) the Amazon basin, (ii) the Andes Mountain Range, and (iii) the Patagonian subregion (Fig. 1A, D).

Distribution modelling indicates greater environmental suitability (>0.7) for *Cochliopsyche* species in broad areas of the NT: (i) the Amazon basin, (ii) the Atlantic Forest domain, and (iii) high-altitude areas in the east of the Caatinga and Cerrado domains (Fig. 1A, C). On the other hand, *Cochliopsyche* areas of low suitability are primarily outside South America (continental Central America and the Lesser and Greater Antilles). In South America, these areas include the Andean, most of the Dry Diagonal (Caatinga, Cerrado, and Chaco domains), and the Patagonian Subregion (Fig. 1C, F).

# Discussion

*Biodiversity knowledge shortfalls and challenges*

Our results demonstrate the clear increment of species descriptions and cataloguing of distributional records, mainly in under-explored areas of the NT. This increase can be related with the development of trichopterology in the NW (as indicated in Holzenthal & Calor 2017; Rasmussen & Morse 2023), cooperation among researchers of NT with other researchers’ groups (e.g., Santos et al. 2020, Calor et al. 2024), and the establishment of research groups in different countries of the NT (e.g., Rueda-Martín & Isa-Miranda 2015; Ríos-Touma et al. 2017, Gama-Neto et al. 2019). After Johanson (Johanson 1995, 1997, 1998, 2002, 2003), who established the modern panorama of Helicopsychidae taxonomy in the NW, several contributions have been made by local researchers (e.g., Bueno-Soria 1983; Rueda-Martín & Isa-Miranda 2015; Dumas & Nessimian, 2019; Moreno et al. 2023; Pereira & Calor 2024), but no one with a broad perspective.

However, our results indicate more dramatic scenarios for *Helicopsyche*, with up to 75% of the fauna still unknown and numerous unexplored areas, when compared with previous estimates for Trichoptera in the NT (Ríos-Touma et al. 2017; Santos et al. 2020, both with around 50% unknown species) (Table 2). These results are consistent when looking at the number of known species and the vast areas with high suitability and little or no research effort, so efforts should be made to describe new species. In addition to unknown species, some valid species exhibit weak circumscription or inconsistencies (see Moreno et al. 2023; Pereira & Calor 2024). In *Cochliopsyche*, there is little difference in genital characteristics (the primary features for species distinction), thus, detailed description of genitalia, standardization, and better circumscription of species are relevant to avoid misidentifications.

The Wallacean shortfall is evident in the limited data available on species distribution (Hortal et al. 2015). Distributional information is often restricted to type localities or adjacent areas (Figs 2A and B), a trend that is also reflected in our results. This highlights the urgent need to increase faunistic surveys in unexplored areas with high environmental suitability (Figs 1C, F, and H) and to analyze the material already housed in collections. Key regions with significant research effort and species richness include: (i) Hispaniola, San Juan, and the Grijalva-Usumacinta freshwater ecoregions in Central America; (ii) the South America Caribbean Drainages—including Trinidad, Maracaibo, Western Amazon Piedmont, and Ucayali-Urubamba Piedmont freshwater ecoregions in northern and western South America; and (iii) parts of the Paraíba do Sul, Northeastern Mata Atlântica, São Francisco, and Fluminense freshwater ecoregions in eastern South America (Fig. 1C).

In contrast, tropical rainforest areas and the dry diagonal regions—including the Caatinga, Cerrado, and Chaco domains—remain largely underexplored despite showing high environmental suitability, indicating strong potential for species distribution. Prioritizing these regions for future faunistic surveys targeting Helicopsychidae is essential to fill existing knowledge gaps. These efforts will likely lead to the discovery of previously undescribed taxa and a more comprehensive understanding of species distributions (Hortal et al. 2015). In doing so, they will help address both Linnean and Wallacean shortfalls, while also contributing to resolving Haeckelian and Hutchinsonian shortfalls if semaphoronts and abiotic conditions are thoroughly documented.

Given the Wallacean and Linnean shortfalls in caddisfly research, our understanding of species diversity and taxonomic groups remains incomplete, particularly within Trichoptera (Calor et al. 2024), where a male-biased focus persists—a gap that aligns with the Haeckelian shortfall (more details, Faria et al. 2021). Biodiversity biases often stem from research concentrated in easily accessible areas with prior survey efforts (Oliveira et al. 2016); however, in this case, the sexual bias is significantly amplified. Therefore, acknowledge that all subsequent discussions are inherently influenced by these knowledge gaps, reflecting the broader discrepancies in our understanding of New World Helicopsychidae, a scenario that is similarly prevalent across most insect orders.

The subgenus *Feropsyche* has 36 valid species, and *Cochliopsyche* comprises ten valid species, all of them with adult females deposited (in material examined), but not formally described (Table 1). Both subgenera have specimens of immature stages deposited in museums and collections that have not yet been associated with adult males (Table 1). These data demonstrate the enormous potential of the material to be described and fulfill the biodiversity shortfalls. The description of these semaphoronts can contribute to a better circumscription of the species, the correction of possible taxonomic errors, and the formation of a complementary database for studies related to the understanding of relationships and biogeographic history (Faria et al. 2021).

To our knowledge, this is the first inference describing environmental gradients for Trichoptera species. Here are addresses Hutchinsonian deficits by empirically defining species' abiotic limits and identifying areas with a higher probability of distribution in regions of high suitability, focusing on the niches of *Feropsyche* and *Cochliopsyche*. This also provides evidence of niche differentiation between the two living lineages of *Helicopsychidae* in the New World. Additionally, these data help identify species more vulnerable to extinction due to their narrow distribution gradients in biogeoclimatic variables. However, we acknowledge that our data are limited by current knowledge of species distribution, and future research may expand the distribution range of species.

*Distribution patterns and potential distribution in New World*

The initial step in addressing the challenges posed by BKS is the compilation of inventories, checklists, and descriptions of new species and new distribution records (Calor et al. 2024). However, it is equally important to consider other aspects, such as ecology, biology, phenology, and ethology, which can inform biodiversity conservation policies and the consequences of anthropogenic interventions (e.g., climate change, suppression of riparian vegetation) (Ríos-Touma et al. 2017; Múrria et al. 2019). This study establishes the “environmental gradients” for the *Feropsyche* and *Cochliopsyche* species. Despite the considerable range of elevation, temperature, and precipitation observed across both subgenera, the data indicated a higher prevalence of *Feropsyche* species in coastal areas of tropical and temperate environments, across a wide elevation gradient (0-5042 m a.s.l.), in low order streams and in areas with high rainfall (Figs 4 and 5). On the other hand, the *Cochliopsyche* species are restricted to tropical environments, with prevalence in rainforests and lowland areas. Of the distributional records, 55% are in higher order streams (>4th order) (Figs 4 and 5).

The results presented here concur with previous hypotheses proposed by Monson et al. (1988), Flint (1974, 1983), and Johanson (2002, 2003). However, this study provides a comprehensive overview of the distribution and analyzes the differences in environmental space between the subgenera. In addition to the morphological differences, these factors contribute to the accumulation of divergence between the NW Helicopsychidae lineages. Currently, there is still no clear resolution regarding their phylogenetic relationship. Some hypotheses suggest that they are sister groups (Johanson et al. 2017), while others posit that they are distant clades (Johanson 1998), further complicating their taxonomic placement.

Despite its wide distribution in tropical regions, *Cochliopsyche* is largely restricted to the type locality or adjacent localities (S1 Table). Distribution records are scarce and generally consist of single collections with a small number of specimens (S1 Table). This is primarily due to the greater concentration of collection and research efforts on Trichoptera in small, oxygenated freshwater environments, where most of the group's diversity is concentrated (Holzenthal et al. 2015). It is therefore recommended that research efforts be directed towards the at inventorying of South America's large rivers, with priority areas for *Cochliopsyche* studies (areas of high suitability and low research effort) including all South America's large river basins (e.g., Amazon, Orinoco, Paraná, and São Francisco River basins), with a particular focus on the large size rivers (Fig. 1C).

The species of *Feropsyche* are distributed across a wide range of habitats, from the temperate areas of the Nearctic Region to the Andean areas of Chile (Fig. 1E). However, the areas with the highest environmental suitability are throughout Central America (including the Antilles), the Florida Keys, areas east of the Andes between Argentina and Bolivia (Chaco domain), and the coastal region of northwest and east South America. Much of continental Central America, the Antilles, and the northwest of South America have been well sampled (Fig. 1D). However, areas of the Chaco domain, the northern portion and Central Corridor of Atlantic Forest, and high-altitude areas of the Cerrado and Caatinga domains remain poorly sampled (Fig. 1E). These areas should be prioritized for research efforts, in agreement with the Brazilian caddisfly studies of Santos et al. (2020), which indicated the to prioritize research efforts in the Northeastern and Central-Western regions of Brazil.

The Patagonian subregion is one of the least researched in the NT, with both groups exhibiting low suitability for this region. However, 11 species of *Feropsyche* are found in areas with minimum temperatures below 0°C (e.g., *H*. (*Feropsyche*) *borealis*, -17°C; *H*. (*Feropsyche*) *minuscula*, -12°C) (Fig. 1D). The data available do not permit us to determine whether the low suitability is (i) due to the absence of favorable environmental conditions, or (ii) to the scarcity of data, which resulted in the model's limitation for this area. In general, there is a considerable range of distribution records for the *Helicopsyche* in the NW.

The species are distributed across a wide environmental gradient, with *Feropsyche* being particularly notable for their ability to occur in various freshwater environments across the New World. This includes a wide range of altitudes, temperatures, and precipitation levels, with a preference for low-order environments (Figs 1, 3, and 4). In contrast, *Cochliopsyche* species typically occur in lowlands, environments with higher average temperatures, and a preference for large rivers, reflecting a restriction in their occurrence to large river basins (Figs 1, 3, and 4). Thus, the results indicate that the two lineages have specialized in different niches: *Feropsyche* specialized in smaller (low-order) environments, while the *Cochliopsyche* specialized in larger (high-order) environments. Similar patterns are also observed in other Trichoptera lineages, including Hydrobiosidae (*Amphichorema* and *Atopsyche*), and Glossosomatidae (*Culoptila* and *Cariboptila*) (Holzenthal & Calor 2017).

The results of this study highlight critical conservation implications for Helicopsychidae species in the New World. The identification of environmental gradients and the discovery of species with restricted distribution in high-suitability and low research effort areas emphasize the need for targeted conservation efforts. Areas with high species richness and environmental suitability, such as parts of Central and South America, should be prioritized for further research and protection. The underexplored regions, especially tropical rainforests and the dry diagonal zones (Caatinga, Cerrado, and Chaco), represent significant gaps in knowledge and potential hotspots for biodiversity. Increasing faunistic surveys in these regions will not only enhance species descriptions but also help mitigate the risk of extinction for species with narrow distribution ranges, which are more susceptible to threats such as climate change and habitat loss. Moreover, the conservation of riparian habitats, particularly in large river basins, is critical to maintaining the integrity of ecosystems supporting these specialized species. By addressing both Linnean, Wallacean, Haeckelian, and Hutchinsonian and other shortfalls, future research can refine conservation strategies and promote the preservation of these important freshwater ecosystems.

**Conclusion**

The results demonstrate recent advances in the taxonomy and cataloguing of distribution records of NW Helicopsychidae, particularly in the under-explored areas of the Neotropical region. Nevertheless, despite the significant increase in the number of species described, there are still numerous gaps in our biodiversity knowledge. It is estimated that up to 75% of the *Helicopsyche* species remain unknown, which underscores the ongoing need for research efforts to describe (preferably with all the semaphoronts) and understand the NW Helicopsychidae fauna. The distribution of *Helicopsyche* species reveals complex patterns influenced by environmental factors such as elevation, temperature, and precipitation. While *Cochliopsyche* species tend to occur in larger river environments, with higher temperatures and a prevalence in extensive river basins, *Feropsyche* species exhibit a high degree of tolerance to different freshwater environments across the Americas, with a prevalence in low-order environments. These distribution patterns reflect the specialization of both subgenera in distinct ecological niches.

Few regions in the New World have been adequately sampled, with most efforts concentrated in areas of historical expeditions (such as the Antilles) or regions with well-established research groups (Central America, the Amazon, and the Atlantic Forest). Due to the high species richness in these areas, they should be prioritized in conservation strategies, with special focus on biodiversity hotspots. However, vast regions remain poorly explored, including the Amazon lowlands, the northern and central areas of the Atlantic Forest, the high-altitude environments of the Caatinga and Cerrado domains, and the transition zones between phytogeographic domains. Research efforts should therefore prioritize these underexplored areas.

This study represents an initial step in developing a more comprehensive database on Trichoptera groups, providing a robust foundation for delineating and understanding biogeographic patterns in the New World. Furthermore, it highlights key biodiversity hotspots and offers tools to address existing knowledge gaps. The compilation presented here significantly enhances the understanding of Helicopsychidae in the New World and may inspire future research proposals for this group or other Trichoptera taxa using similar approaches.

# Acknowledgements

We thank the Instituto Chico Mendes de Conservação da Biodiversidade (ICMBio) for collecting permits. ARC acknowledge the Conselho Nacional de Desenvolvimento Científico e Tecnológico (CNPq) (under Grants 303623/2015-2). RP also thanks to the Coordenação de Aperfeiçoamento de Pessoal de Nível Superior (CAPES, finance code 001, PDS-CAPES-88882.453922/2019-01, and PRAPG-CAPES-88887.986811/2024-00) for the doctoral and post-doctoral fellowship. We thank the Conselho Nacional de Desenvolvimento Científico e Tecnológico (CNPq), Programa de Apoio à Pós-Graduação (PROAP-CAPES) for financial support, and this study was financed in part by the CAPES – Finance Code 001 (PPG Biodiversidade e Evolução). We would like to thank Dra. Neusa Hamada, Dra. Ana Pes and Dr. Gleison Desidério for providing us with data from LACIA-INPA.

**Conflict of interest**

The authors declare that they have no conflict of interest.

# References

Abell R, et al (2008) Freshwater ecoregions of the world: a new map of biogeographic units for freshwater biodiversity conservation. BioScience 58:403–414. https://doi.org/10.1641/B580507

Brauer F (1865) Zweiter Bericht über auf der Weltfart der Kais Fregatte Novara gesammelten Neuropteren. Verhandlungen der Zoologisch-Botanischen Gesellschaft in Wien 15:415–422.

Bueno-Soria J (1983) Five new species of caddisflies (Trichoptera) from Mexico. Proceedings of the Entomological Society of Washington 85:450–455.

Calor AR, Amorim DS, Holzenthal RW (2006) Phylogenetic analysis of *Notalina* (*Neonotalina*), with descriptions of two new species. Zootaxa 1131:33–48. https://doi.org/10.11646/zootaxa.1131.1.2

Calor AR, et al (2023) Aquatic Entomology Lab “Prof. Cláudio Gilberto Froehlich” and the task of facing the biodiversity knowledge deficits on caddisflies (Trichoptera), Bahia, Brazil. Revista Brasileira de Entomologia 67(esp.), e20230065. https://doi.org/10.1590/1806-9665-RBENT-2023-0065

Carpenter G, Gillison AN, Winter J (1993) DOMAIN: a flexible modelling procedure for mapping potential distributions of plants and animals. Biodiversity and Conservation 2:667–680.

Cavalcante-Silva A, Pereira R, Calor AR (2022) Caddisflies (Trichoptera) checklist and a new species of Helicopsyche von Siebold, 1856, from the Brejo de Altitude de Triunfo, a relict rainforest within the Caatinga domain, Northeast Brazil. ZooKeys 1111:215–244. https://doi.org/10.3897/zookeys.1111.77541

Ceballos G, Ehrlich PR, Dirzo R (2017) Biological annihilation via the ongoing sixth mass extinction signaled by vertebrate population losses and declines. Proceedings of the National Academy of Sciences of the United States of America 114: e6089. https://doi.org/10.1073/pnas.1704949114

Collen B, Ram M, Zamin T, McRae L (2008) The tropical biodiversity data gap: addressing disparity in global monitoring. Tropical Conservation Science 1:75–88.

de Moor FC, Ivanov VD (2008) Global diversity of caddisflies (Trichoptera: Insecta) in freshwater. Hydrobiologia 595:393–407. https://doi.org/10.1007/s10750-007-9113-2

Desiderio GR, Santana V, Pereira ES, Pes AM, Hamada N (2021) On the identity of *Smicridea* (*Smicridea*) *aequalis* Banks, 1920 (Trichoptera: Hydropsychidae): Morphology of adults and immature stages, bionomics, distribution, and male color dimorphism. Neotropical Entomology 50:430–443. https://doi.org/10.1007/s13744-021-00860-8

Droissart V, et al (2018) Beyond trees: biogeographical regionalization of tropical Africa. Journal of Biogeography 45:1153–1167. https://doi.org/10.1111/jbi.13190

Dumas LL, Nessimian JL (2019) New species of *Helicopsyche* von Siebold 1856 (Trichoptera: Helicopsychidae) from Brazil, including the redescription of *Helicopsyche* (*Feropsyche*) *planorboides* Machado 1957. Zootaxa 4619:231–250. https://doi.org/10.11646/zootaxa.4619.2.2

Ely CV, Bordignon SAL, Trevisan R (2017) Implications of poor taxonomy in conservation. Journal for Nature Conservation 36:10–13. https://doi.org/10.1016/j.jnc.2017.01.003

Faria LRR, Pie MR, Salles FF, Soares EDG (2021) The Haeckelian shortfall or the tale of the missing semaphoronts. Journal of Zoological Systematics and Evolutionary Research 59:359–369.

Flint OS Jr (1972) Studies of Neotropical caddisflies, XIV: on a collection from northern Argentina. Proceedings of the Biological Society of Washington 85:223–248.

Flint OS Jr (1974) Checklist of Trichoptera, or caddisflies, of Chile. Revista Chilena de Entomología 8:83–93.

Flint OS Jr (1983) Studies of Neotropical caddisflies, XXXIII: new species from Austral South America (Trichoptera). Smithsonian Contributions to Zoology 377:1–98.

Flint OS Jr (1986) Studies of Neotropical caddisflies, XXXVI. The genus *Cochliopsyche* in Middle America (Trichoptera: Helicopsychidae). Anales del Instituto de Biología, Universidad Nacional Autónoma de México, Series de Zoología 56:213–216.

Gama-Neto JL, Ribeiro JMF, Passos MAB (2019) Two new species of *Helicopsyche* Siebold 1856 (Insecta: Trichoptera: Helicopsychidae) from Pará State, northern Brazil. Zootaxa 4565:420–426. http://doi.org/10.11646/zootaxa.4565.3.8

Hagen HA (1861) Synopsis of the Neuroptera of North America with a list of the South American species. Smithsonian Institution Miscellaneous Collections 4:1–347. https://doi.org/10.5962/bhl.title.60275.

Hagen HA (1964) Ueber Phryganiden-Gehäuse. Stettiner Entomologische Zeitung 25:113–116.

Hamilton SW (1982) Historical biogeography of two groups of Caribbean *Polycentropus* (Trichoptera: Polycentropodidae). In: Liebherr JK (Ed.), Zoogeography of Caribbean Insects (pp. 153–182). Ithaca: Cornell University Press.

Hanley JA, McNeil BJ (1982) The meaning and use of the area under a receiver operating characteristic (ROC) curve. Radiology 143:29–36.

Harper M, et al (2021) Twenty-five essential research questions to inform the protection and restoration of freshwater biodiversity. Aquatic Conservation: Marine and Freshwater Ecosystems 31:2632–2653. https://doi.org/10.1002/aqc.3634

Harvey BJ, et al (2023) Emergent hotspots of biotic disturbances and their consequences for forest resilience. Frontiers in Ecology and the Environment 21(8):388–396.

Holzenthal RW (1986) The Neotropical species of *Notalina*, a southern group of long-horned caddisflies (Trichoptera: Leptoceridae). Systematic Entomology 11:61–73. https://doi.org/10.1111/j.1365-3113.1986.tb00165.x

Holzenthal RW, Calor AR (2017) Catalog of the Neotropical Trichoptera (Caddisflies). ZooKeys 654:1–566. https://doi.org/10.3897/zookeys.654.9516.

Holzenthal RW, Thomson RE, Ríos-Touma B (2015) Order Trichoptera. In: Thorp JH, Covich AP (Eds.), Thorp and Covich's Freshwater Invertebrates (pp. 965–1002). Academic Press.

Hortal J, Borges PAV, Gaspar C (2006) Evaluating the performance of species richness estimators: sensitivity to sample grain size. Journal of Animal Ecology 75:274–287. http://doi.org/10.1111/j.1365-2656.2006.01048.x

Hortal J, Bello F, Diniz-Filho JAF, Lewinsohn TM, Lobo JM, Ladle RJ (2015) Seven shortfalls that beset large-scale knowledge of biodiversity. Annual Review of Ecology, Evolution, and Systematics 46:523–549. https://doi.org/10.1146/annurev-ecolsys-112414-054400

VanDerWal J, Shoo LP, Graham C, Williams SE (2009) Selecting pseudo-absence data for presence-only distribution modeling: how far should you stray from what you know? Ecological Modelling 220:589–594. https://doi.org/10.1016/j.ecolmodel.2008.11.010

Johanson KA (1995) A catalog of the Helicopsychidae (Insecta: Trichoptera) of the world. Bulletin of Zoological Museum 14:101–123.

Johanson KA (1997) Zoogeography and diversity of the snail case caddisflies (Trichoptera: Helicopsychidae). In: Holzenthal RW, Flint Jr OS (Eds), Proceedings of the 8th International Symposium on Trichoptera, pp. 205–212. Columbus, Ohio: Ohio Biological Survey.

Johanson KA (1998) Phylogenetic and biogeographic analysis of the family Helicopsychidae (Insecta: Trichoptera). Entomologica Scandinavica Supplement 53:1–172.

Johanson KA (2002) Systematic revision of American *Helicopsyche* of subgenus *Feropsyche* (Trichoptera: Helicopsychidae). Insect Systematics & Evolution 60:1–151.

Johanson KA (2003) Revision of the Neotropical caddisfly subgenus *Helicopsyche* (*Cochliopsyche*) (Trichoptera, Helicopsychidae), with descriptions of twelve new species. Insect Systematics & Evolution 34:381–414.

Johanson KA, Malm T, Espeland M (2017) Molecular phylogeny of Sericostomatoidea (Trichoptera) with the establishment of three new families. Systematic Entomology 42:240–266. https://doi.org/10.1111/syen.12209.

Kier G, Mutke J, Dinerstein E, Ricketts TH, Küper W, Kreft H, Barthlott W (2005) Global patterns of plant diversity and floristic knowledge. Journal of Biogeography 32:1–10. https://doi.org/10.1111/j.1365-2699.2005.01272.x.

Kindt R, Coe R (2005) Tree diversity analysis: A manual and software for common statistical methods for ecological and biodiversity studies. Nairobi: World Agroforestry Centre (ICRAF).

Kreft H, Jetz W (2010) A framework for delineating biogeographical regions based on species distributions. Journal of Biogeography 37:2029–2053. https://doi.org/10.1111/j.1365-2699.2010.02375.x.

Kusch J (2015) Interacting influences of climate factors and land cover types on the distribution of caddisflies (Trichoptera) in streams of a central European low mountain range. Insect Conservation and Diversity 8(1):92–101.

Larsson M, Göthberg A, Milberg P (2020) Night, light and flight: light attraction in Trichoptera. Insect Conservation and Diversity 13(3):296–302.

McFarlane AG (1973) Five new species of Trichoptera from New Zealand. Journal of the Royal Society of New Zealand 3:23–34.

Monson MP, Holzenthal RW, Ahlstrand GG (1988) The larva and pupa of *Cochliopsyche vazquezae* (Trichoptera: Helicopsychidae). Journal of the North American Benthological Society 7:152–159.

Montoya JM, Donohue I, Pimm SL (2018) Planetary gradients for biodiversity: implausible science, pernicious policies. Trends in Ecology & Evolution 33:71–73. https://doi.org/10.1016/j.tree.2017.10.004.

Moreno L, Desidério GR, Pes AMO, Hamada N (2023) Two new species of *Helicopsyche* (*Feropsyche*) Johanson (Trichoptera: Helicopsychidae) from Central Amazon, Brazil. Annals of the Entomological Society of America 73:29–40. https://doi.org/10.3161/00034541ANZ2023.73.1.003.

Morrone JJ (2014) Biogeographical regionalization of the Neotropical region. Zootaxa 3782:1–110. https://doi.org/10.11646/zootaxa.3782.1.1

Morse JC (2017) Biodiversity of aquatic insects. Insect Biodiversity Science and Society 1:205–227. https://doi.org/10.1002/9781118945568

Morse JC (2024) Trichoptera World Checklist. http://entweb.clemson.edu/database/trichopt/index.htm. Accessed 24 September 2024

Morse JC, Frandsen PB, Graf W, Thomas JA (2019) Diversity and Ecosystem Services of Trichoptera. Insects 10:100–125. https://doi.org/10.3390/insects10050125

Müller F (1885) Wie Entsteht die Gleiderung der Insektenfuhler? Kosmos 17:201–204.

Nelder JA, Wedderburn RWM (1972) Generalized linear models. Journal of the Royal Statistical Society: Series A (General) 135:370–384.

Múrria C, Sáinz‐Bariáin M, Vogler AP, Viza A, González M, Zamora‐Muñoz C (2020) Vulnerability to climate change for two endemic high‐elevation, low‐dispersive *Annitella* species (Trichoptera) in Sierra Nevada, the southernmost high mountain in Europe. Insect Conservation and Diversity 13(3):283-295.

Nix HA (1986) A biogeographic analysis of Australian elapid snakes. Atlas of Elapid Snakes of Australia. Canberra: Australian Gov. Publ. Service.

Oksanen J et al. (2019) Vegan: Community Ecology Package. Retrieved from https://cran.r-project.org/web/packages/vegan.

Oláh J, Oláh J Jr (2022) New Neotropical Trichoptera: *Banyallarga* and *Phylloicus* species delineation by revisited paraproct (Calamoceratidae). Opuscula Zoologica (Budapest) 53:205–225. https://doi.org/10.18348/opzool.2022.2.205.

Oliveira U et al. (2016) The strong influence of collection bias on biodiversity knowledge shortfalls of Brazilian terrestrial biodiversity. Diversity and Distributions 22:1232–1244. https://doi.org/10.1111/ddi.12489.

Palmer MW (1990) The estimation of species richness by extrapolation. Ecology 71:1195–1198. https://doi.org/10.2307/1937387.

Pereira R, Calor AR (2024) *Helicopsyche* (*Feropsyche*) Johanson, 1998 (Trichoptera) from Northeastern Mata Atlântica Freshwater ecoregion: integrating taxonomy and niche modelling. Anais da Academia Brasileira de Ciências 96:e20230369. https://doi.org/10.1590/0001-3765202420230369.

Pes AM, Holzenthal RW, Sganga JV, Santos APM, Barcelos-Silva P, Camargos LM (2018) Order Trichoptera. In: Hamada N, Thorp JD, Rogers C (Eds), Thorp and Covich’s Freshwater Invertebrates. 4th Edition. Vol. 3. Keys to Neotropical Hexapoda (pp. 237–324). San Diego: Academic Press. https://doi.org/10.1016/b978-0-12-804223-6.00010-x.

Rasmussen AK, Morse JC (2024) Distributional Checklist of Nearctic Trichoptera (2022 Revision). Florida A&M University, Tallahassee. http://www.Trichoptera.org. Accessed 5 September 2024.

Razo-González M, Novelo-Gutiérrez R, Castaño-Meneses G, Márquez J (2023) Diversity and composition of caddisflies (Insecta: Trichoptera) along an elevation gradient in southeastern Mexico. Diversity 15(1):110.

Ríos-Touma B, Holzenthal RW, Huisman J, Thomson R, Rázuri-Gonzales E (2017) Diversity and distribution of the caddisflies (Insecta: Trichoptera) of Ecuador. PeerJ 5:e2851. https://doi.org/10.7717/peerj.2851.

Ríos-Touma B, Cuesta F, Rázuri-Gonzales E, Holzenthal R, Tapia A, Calderón-Loor M (2022) Elevational biodiversity gradients in the Neotropics: Perspectives from freshwater caddisflies (Insecta: Trichoptera). PloS One 17(8):e0272229. https://doi.org/10.1371/journal.pone.0272229

Robertson DR, Holzenthal RW (2005) The Neotropical caddisfly genus *Tolhuaca* (Trichoptera: Glossosomatidae). Zootaxa 1063:53–68.

Ross HH, King EW (1952) Biogeographic and taxonomic studies in *Atopsyche* (Trichoptera, Rhyacophilidae). Annals of the Entomological Society of America 45:177–204. https://doi.org/10.1093/aesa/45.2.177

Rosvall M, Bergstrom C (2008) Maps of information flow reveal community structure in complex. PNAS 105:1118–1123. https://doi.org/10.1073/pnas.0706851105

Rueda-Martín PA, Isa-Miranda AV (2015) Association of immature stages of some caddisfly species from northwestern Argentina with description of a new species of Helicopsyche (Trichoptera: Helicopsychidae). Zootaxa 3949:203–216. https://doi.org/10.11646/zootaxa.3949.2.3

Sánchez-Bayo F, Wyckhuys KA (2019) Worldwide decline of the entomofauna: A review of its drivers. Biological Conservation 232:8-27. https://doi.org/10.1016/j.biocon.2019.01.020

Santos APM, Dumas LL, Henriques-Oliveira AL, Souza WRM, Camargos LM, Calor AR, Pes AMO (2020) Taxonomic Catalog of the Brazilian Fauna: order Trichoptera (Insecta), diversity and distribution. Zoologia (Curitiba) 37:e46392. https://doi.org/10.3897/zoologia.37.e46392

Souza WRM, Lima LRC, Pes, AMO, Pinheiro U (2013a). Trichoptera (Insecta) from Pernambuco state, northeastern Brazil. Journal of Natural History 47: 1–10. https://doi.org/10.1080/00222933.2013.791948

Strayer DL, Dudgeon D (2010) Freshwater biodiversity conservation: recent progress and future challenges. Journal of the North American Benthological Society 29:344–358. https://doi.org/10.1899/08-171.1

Swainson W (1840) A Treatise on Malacology; or the Natural Classification of Shells and Shellfish. Leipzig: Wilhelm Engelmann

Tax D, Duin R (2004) Support Vector Data Description. Machine Learning 54:45–66. http://doi.org/10.1023/B:MACH.0000008084.60811.49

Terribile LC, Feitosa DT, Pires MG, de Almeida PCR, de Oliveira G, Diniz-Filho JAF et al. (2018) Reducing Wallacean shortfalls for the coral snakes of the *Micrurus lemniscatus* species complex: Present and future distributions under a changing climate. PlosOne 13:e0205164. https://doi.org/10.1371/journal.pone.0205164

Tessarolo G et al. (2021) Using maps of biogeographical ignorance to reveal the uncertainty in distributional data hidden in species distribution models. Ecography 44:1743–1755. doi.org/10.1111/ecog.05793

Thomas JA, Frandsen PB, Prendini E, Zhou X, Holzenthal RW (2020) A multigene phylogeny and timeline for Trichoptera (Insecta). Systematic Entomology 45:670–686. https://doi.org/10.1111/syen.12422

Thomas JA, Frandsen PB, Morse JC (2023) Revised chronology of Trichoptera evolution. Contributions to Entomology 72:289–294

Tickner D, Opperman JJ, Abell R, Acreman M, Arthington AH, Bunn SE, Cooke SJ et al. (2020) Bending the Curve of Global Freshwater Biodiversity Loss: An Emergency Recovery Plan. BioScience 70:330–342. https://doi.org/10.1093/biosci/biaa002

Ulmer G (1905) Zur Kenntniss aussereuropäischer Trichopteren. (Neue Trichoptern des Hamburger und Stettiner Museums und des Zoologischen Instituts in Halle, nebst Beschreibungen einiger Typen Kolenati’s und Burmeister’s.). Stettiner Entomologische Zeitung 66:1–119

Ulmer G (1910) Trichoptera. in: Percy Sladen Trust Expedition, Indian Ocean in 1905. Transactions of the Linnean Society of London, 2nd Series, 14:41–54

Vallot JN (1855) Sur deux fourreaux hélicoïdes façonnés par les larves d’insectes. Mémoires de l’Académie des Sciences, Arts et Belles-Lettres de Dijon

Vinson MR, Hawkins CP (2003) Broad-scale geographical patterns in local stream insect genera richness. Ecography 26(6):751–767

von Siebold CTE (1856) Wahre Parthenogenesis bei Schmetterlingen und Bienen. Ein Beitrag zur Fortpflanzung Geschichte der Thiere

Wallace AR (1876) The Geographical Distribution of Animals. Cambridge University Press, Cambridge, UK

Watson JEM et al. (2018) The exceptional value of intact forest ecosystems. Nature Ecology & Evolution 2:599–610. https://doi.org/10.1038/s41559-018-0490-x

Yang W, Ma K, Kreft H (2013) Geographical sampling bias in a large distributional database and its effects on species richness-environment models. Journal of Biogeography 40:1415–1426. https://doi.org/10.1111/jbi.12108

**Tables**

**Table 1.** Species of *Helicopsyche* (*Feropsyche*) and information of known semaphoronts, distribution, and collections with deposited material. KS = Known semaphoronts; ♂ = Male; ♀ = Female; L = Larvae; P = Pupa; Collections that house type material in bold; *semaphoronts present in material examined of the publications, but not formally described.

| **Species** | **KS** | **Museum** | **Distribution** |
| --- | --- | --- | --- |
| *H*. (*C*.) *amazona* Johanson, 2003 | ♂ | **USNM**, NRM | BRA |
| *H*. (*C*.) *amica* Johanson, 2003 | ♂, ♀* | MVC, USNM, **MCZ**, ISNB | BRA, GUY, VEN |
| *H*. (*C*.) *blahniki* Johanson, 2003 | ♂, ♀* | **UMSP**, CIUC, FMNH, MVC, USNM NRM | BRA, COL, ECU, GUY, PER, VEN |
| *H*. (*C*.) *brazilia* Johanson, 2003 | ♂, ♀* | **MZUSP**, USNM, MRM | BRA |
| *H*. (*C*.) *chocoensis* Johanson, 2003 | ♂, ♀* | **USNM** | BRA, COL |
| *H*. (*C*.) *clara* (Ulmer, 1905) | ♂, ♀ | **MCZ**, MZUSP, UMSP, USNM | ARG, BRA, ECU |
| *H*. (*C*.) *holzenthali* Johanson, 2003 | ♂, ♀* | **UMSP**, USMN | VEN |
| *H*. (*C*.) *lobata* (Flint, 1983) | ♂ | **USNM**, MCZ, MZUSP, UMSP | ARG, BRA, PER |
| *H*. (*C*.) *nyurga* Oláh & Oláh, 2022 | ♂ | **OPC** | ECU |
| *H*. (*C*.) *napoa* Johanson, 2003 | ♂, ♀* | **USNM** | ECU |
| *H*. (*C*.) *ocosingua* Johanson, 2003 | ♂, ♀* | **INHS**, NRM | BRA, MEX |
| *H*. (*C*.) *opalescens* (Flint, 1972) | ♂ | **USNM**, FNMH, MZUSP, UMSP | ARG, BRA, ECU, GUY, PAR, PER, SUR, URU, VEN |
| *H*. (*C*.) *pandeirosa* Johanson, 2003 | ♂, ♀* | **MZUSP**, UMSP, NRM, USNM | BRA |
| *H*. (*C*.) *paraguaiensis* Johanson, 2003 | ♂ | **USNM** | PAR |
| *H*. (*C*.) *puyoa* Johanson, 2003 | ♂, ♀* | **USNM**, UMSP | BRA, ECU |
| *H*. (*C*.) *vazquezae* (Flint, 1986) | ♂, L, P, C | **USNM**, INHS, UMSP | BOL, COR, ECU, MEX, VEN |
| *H*. (*C*.) *xinguensis* Johanson, 2003 | ♂, ♀* | **MZUSP**, USNM, UMSP | BRA |
| *H*. (*F*.) *alajuela* Johanson & Holzenthal, 2010 | ♂ | **NMNH;** COZEM | CRI; PAN |
| *H*. (*F*.) *altercoma* Botosaneanu & Flint, 1991 | ♂♀ | **NMNH**; CMNH; FSCA; ZMUA | DOM |
| *H*. (*F*.) *angeloi* Holzenthal, Blahnik & Calor, 2016 | ♂♀* | **MZUSP**; UFBA; UMSP | BRA |
| *H*. (*F*.) *angulata* Flint, 1981 | ♂♀ | **USNM**; UMSP; NRM | COL; ECU; VEN |
| *H*. (*F*.) *apicauda* Flint, 1968 | all | **USNM;** NMNH | DMA; GUA |
| *H*. (*F*.) *auroa* Johanson & Holzenthal, 2004 | ♂♀* | **UMSP**; NMNH | VEN |
| *H*. (*F*.) *bendego* Dumas & Nessimian, 2019 | ♂♀* | **DZRJ** | BRA |
| *H*. (*F*.) *blancasi* Schmid, 1958 | ♂♀* | **NMNH** | PER |
| *H*. (*F*.) *blantoni* Johanson & Malm, 2006 | ♂ | **NMNH;** NRM | PAN |
| *H*. (*F*.) *borealis* (Hagen, 1861) | all | **MCZ**; UMSP; NMNH; TAMU; USNM | CAN; CRI; GTM; HND; MEX; NIC; PAN; USA |
| *H*. (*F*.) *braziliensis* (Swainson, 1840) | C | - | BRA |
| *H*. (*F*.) *breviterga* Flint, 1991 | ♂♀* | **UMSP**; COZEM; UMSP; NRM | COL; PAN; VEN |
| *H*. (*F*.) *caligata* Flint, 1967 | ♂ | **NMNH** | CHI |
| *H*. (*F*.) *camuriensis* Johanson & Holzenthal, 2004 | ♂♀* | **UMSP** | VEN |
| *H*. (*F*.) *carajas* Gama Neto, Ribeiro & Passos, 2019 | ♂ | **MPEG** | BRA |
| *H*. (*F*.) *catoles* Souza, Gomes & Calor, 2017 | ♂♀* | **MZUSP**; UFBA; UFRJ | BRA |
| *H*. (*F*.) *centrocubana* Botosaneanu & Flint, 1991 | ♂L | **ZMUA** | CUB |
| *H*. (*F*.) *chilensis* Flint, 1983 | ♂♀* | **NMNH** | CHI |
| *H*. (*F*.) *chiriquensis* Johanson & Malm, 2006 | ♂♀* | **NMNH**; UMSP; INBIO | CRI; PAN |
| *H*. (*F*.) *cipoensis* Johanson & Malm, 2006 | ♂ | **NMNH** | BRA |
| *H*. (*F*.) *circulata* Johanson & Holzenthal, 2004 | ♂ | **UMSP** | VEN |
| *H*. (*F*.) *cochleara* Johanson, 1999 | ♂ | **NMNH** | ECU |
| *H*. (*F*.) *colombiensis* von Siebold, 1856 | C | **-** | COL; VEN |
| *H*. (*F*.) *comosa* Kingsolver, 1964 | ♂♀ | **INHS**; ZMUA; NMNH; MCZ | CUB |
| *H*. (*F*.) *cotopaxi* Botosaneanu & Flint, 1982 | ♂♀*L*P*C | **USNM**; ZMUA | ECU |
| *H*. (*F*.) *cubana* Kingsolver, 1964 | all* | **INHS**; NHMJ; ZMUA | CUB; JAM |
| *H*. (*F*.) *curvipalpia* Johanson & Malm, 2006 | ♂♀* | **INHS**; NRM | MEX |
| *H*. (*F*.) *dampfi* Ross, 1956 | ♂♀*P* | **INHS**; CNHM; NMNH; UMSP; INBIO; MEL | CRI; GTM; MEX; NIC |
| *H*. (*F*.) *daome* Dumas & Nessimian, 2019 | ♂♀* | **DZRJ** | BRA |
| *H*. (*F*.) *diamantina* Pereira & Calor, 2024 | ♂ | **MZUSP; UFBA** | BRA |
| *H*. (*F*.) *dinoprata* Dumas & Nessimian, 2019 | ♂♀* | **DZRJ**; MZUSP | BRA |
| *H*. (*F*.) *disjuncta* Johanson & Holzenthal, 2004 | ♂ | **NMNH** | VEN |
| *H*. (*F*.) *dominicana* Botosaneanu & Flint, 1991 | ♂♀ | **USNM; CMNH; CNHM; FSCA; NMNH; ZMUA** | DOM |
| *H*. (*F*.) *dorsocurvata* Johanson & Holzenthal, 2010 | ♂ | **UMSP** | CRI |
| † *H*. (*F*.) *electra* Johanson & Wichard, 1996 | ♂ | Collection Wichard | DOM |
| *H*. (*F*.) *extensa* Ross, 1956 | ♂♀* | **INHS**; UMSP | PER; VEN |
| *H*. (*F*.) *falcigona* Botosaneanu & Flint, 1991 | ♂♀*L | **ZMUA**; USNM; MCZ | CUB |
| *H*. (*F*.) *fistulata* Flint, 1991 | ♂♀* | **USNM** | COL; VEN |
| *H*. (*F*.) *flinti* Johanson, 1999 | ♂ | **BMNH** | BRA |
| *H*. (*F*.) *fridae* Johanson, 2003 | ♂♀* | **NMNH**; UCD | PAN |
| *H*. (*F*.) *golfitoensis* Johanson & Holzenthal, 2010 | ♂ | **NMNH** | CRI |
| *H*. (*F*.) *granpiedrana* Botosaneanu & Sykora, 1973 | ♂ | **ZMUA** | CUB |
| *H*. (*F*.) *grenadensis* Flint & Sykora, 1993 | ♂♀* | **FSCA**; NMNH; UMSP; NRM | GRE; VEN |
| *H*. (*F*.) *guadeloupensis* Malicky, 1980 | all | **CM**; ZMUA; MHNH; CMNH; MNNM; MNHM | DMA; GUA; LCA; MTQ |
| *H*. (*F*.) *guara* Holzenthal, Blahnik & Calor, 2016 | ♂♀* | **MZUSP**; UMSP; UFBA | BRA |
| *H*. (*F*.) *guariru* Vilarino & Calor, 2017 | ♂ | **MZUSP**; UMSP; UFBA | BRA |
| *H*. (*F*.) *hageni* Banks, 1938 | all | **MCZ**; ZMUA | CUB; DOM |
| *H*. (*F*.) *haitiensis* Banks, 1938 | ♂ | **MCZ** | HTI |
| *H*. (*F*.) *helicoidella* (Vallot, 1855) | C | - | BRA |
| *H*. (*F*.) *incisa* Ross, 1956 | ♂♀* | **INHS**; UMSP; INBIO; NMNH; NRM | CRI; MEX; NIC; PAN |
| *H*. (*F*.) *imperial* Silva-Pereira, Desidério, Pereira, Hamada, 2024 | ♂ | **INPA, UFBA** | BRA |
| *H*. (*F*.) *johansoni* Moreno, Desidério, Pes & Hamada, 2023 | ♂ | **INPA; DZRJ; MNRJ; UFBA** | BRA |
| *H*. (*F*.) *inflata* Gama Neto, Ribeiro & Passos, 2019 | ♂ | **MPEG** | BRA |
| *H*. (*F*.) *kalaom* Botosaneanu, 1996 | ♂♀* | **ZMUA**; FSCA; NMNH; CMNH | DOM |
| *H*. (*F*.) *kingstona* Johanson, 2003 | ♂ | **UCD** | JAM |
| *H*. (*F*.) *krenak* Bonfá-Neto, Vilarino & Salles, 2023 | ♂ | **UFVB** | BRA |
| *H*. (*F*.) *lambda* Flint, 1983 | ♂ | **NMNH** | ARG |
| *H*. (*F*.) *laneblina* Johanson & Holzenthal, 2004 | ♂ | **NMNH** | VEN |
| *H*. (*F*.) *lara* Johanson & Holzenthal, 2004 | ♂♀* | **UMSP**; IZAM; NRM | VEN |
| *H*. (*F*.) *lazzariae* Holzenthal, Blahnik & Calor, 2016 | ♂ | **MZUSP** | BRA |
| *H*. (*F*.) *lewalleni* Denning & Blickle, 1979 | ♂♀ | **CAS**; INBIO; UMSP | CRI; ELS |
| *H*. (*F*.) *limnella* Ross, 1937 | ♂ | **INHS** | **USA** |
| *H*. (*F*.) *linabena* Johanson & Holzenthal, 2004 | ♂♀* | **NMNH** | VEN |
| *H*. (*F*.) *linguata* Johanson & Malm, 2006 | ♂ | **NMNH** | PAN |
| *H*. (*F*.) *lutea* (Hagen, 1961) | ♀ | **MCZ** | DOM |
| *H*. (*F*.) *luziae* 17 | ♂♀* | **DZRJ** | BRA |
| *H*. (*F*.) *maculisternum* Botosaneanu, 1993 | ♂♀* | **ZMUA** | VEN; TRI |
| *H*. (*F*.) *manaos* Moreno, Desidério, Pes & Hamada, 2023 | ♂ | **NMNH**; ZMUA; CMNH; FSCA | DOM |
| *H*. (*F*.) *melanochaeta* Flint & Sykora, 2004 | ♂♀* | **INPA; DZRJ; MNRJ; UFBA** | BRA |
| *H*. (*F*.) *merida* Botosaneanu & Flint, 1982 | ♂♀*L*P*C | **NMNH**; ZMUA; UMSP | VEN |
| *H*. (*F*.) *mexicana* Banks, 1901 | ♂♀ | **MCZ;** INHS; NMNH; USNM; CAS; OSU; UCR | CRI; MEX; USA |
| *H*. (*F*.) *minima* von Siebold, 1856 | all | **USNM**; ZMUA | NIC; PRI |
| *H*. (*F*.) *minuscula* Martynov, 1912 | ♀ | **PAN** | PER |
| *H*. (*F*.) *molesta* Botosaneanu, 1998 | ♂ | **ZMUA** | JAM |
| *H*. (*F*.) *mateusi* Pereira & Calor, 2024 | ♂ | **MZUSP; UFBA** | BRA |
| *H*. (*F*.) *miltonsantosi* Pereira & Calor, 2024 | ♂ | **MZUSP; UFBA** | BRA |
| *H*. (*F*.) *monda* Flint, 1983 | ♂ | **USNM**; DZRJ; NMNH | ARG; BRA; PRY; VEN |
| *H*. (*F*.) *montana* Felber, 1912 | LPC | **NMB** | MEX |
| *H*. (*F*.) *muelleri* Banks, 1920 | ♂♀*LPC | **MCZ**; IRSNB | ARG; BRA; PER |
| *H*. (*F*.) *neblinensis* Johanson & Holzenthal, 2004 | ♂♀* | **NMNH**; IZAM; NRM | VEN |
| *H*. (*F*.) *nigrisensilla* Botosaneanu & Flint. 1991 | ♂♀ | **USNM; ZMUA** | DOM |
| *H*. (*F*.) *obscura* Rueda Martín & Isa Miranda, 2015 | ♂LPC | **IBN** | ARG |
| *H*. (*F*.) *occidentale* Botosaneanu & Flint, 1991 | ♂L | **USNM; ZMUA** | CUB |
| *H*. (*F*.) *ochthephila* Flint, 1968 | all | **NMNH** | JAM |
| *H*. (*F*.) *paprockii* Johanson & Malm, 2006 | ♂ | **NMNH** | BRA |
| *H*. (*F*.) *parahageni* Flint & Sykora, 2004 | ♂♀ | **NMNH;** CMNH; FSCA | DOM |
| *H*. (*F*.) *paralimnella* Hamilton, 1989 | ♂ | **CUEC** | USA |
| *H*. (*F*.) *paucispina* Botosaneanu & Flint, 1991 | ♂ | **ZMUA** | CUB |
| *H*. (*F*.) *perija* Johanson & Holzenthal, 2004 | ♂ | **UMSP** | VEN |
| *H*. (*F*.) *peruana* Banks, 1920 | ♂ | **MCZ** | PER |
| *H*. (*F*.) *paulofreirei* Pereira & Calor, 2024 | ♂ | **MZUSP; UFBA** | BRA |
| *H*. (*F*.) *petri* Dumas & Nessimian, 2019 | ♂ | **DZRJ; MZUSP** | BRA |
| *H*. (*F*.) *pietia* Denning, 1964 | ♂♀ | **CAS**; INHS; NRM | MEX; USA |
| *H*. (*F*.) *piroa* Ross, 1944 | ♂♀* | **INHS**; TAMU; USNM | CRI; MEX; NIC; USA |
| *H*. (*F*.) *planata* Ross, 1956 | ♂ | **INHS**; CNIN; CUEC | NIC; MEX |
| *H*. (*F*.) *planorboides* Machado, 1957 | all | **DZRJ** | BRA |
| *H*. (*F*.) *poliochaeta* Flint & Sykora, 2004 | ♂♀ | **NMNH**; FSCA | DOM |
| *H*. (*F*.) *propinqua* Botosaneanu & Flint, 1991 | ♂ | **NMNH** | PRI |
| *H*. (*F*.) *quadrosa* Ross, 1956 | ♂ | **INHS** | MEX |
| *H*. (*F*.) *ralphi* Cavalcante-Silva, Pereira & Calor, 2022 | all | **MZUSP**; INPA; UFBA; UFRJ | BRA |
| *H*. (*F*.) *ramosi* Flint, 1964 | all | **NMNH**; ZMUA | PRI |
| *H*. (*F*.) *rentzi* Denning & Blickle, 1979 | ♂♀ | **CAS**; INBIO; UMSP; USNM | CRI |
| *H*. (*F*.) *sanblasensis* Johanson & Malm, 2006 | ♂ | **NMNH** | PAN |
| *H*. (*F*.) *scalaris* Hagen, 1864 | C | - | VEN |
| † *H*. (*F*.) *electra* Johanson & Wichard, 1996 | ♂ | **Collection Wichard** | DOM |
| *H*. (*F*.) *selanderi* Ross, 1956 | ♂♀* | **INHS**; NMNH; UMSP | CRI; MEX; VEN |
| *H*. (*F*.) *septifera* Flint & Sykora, 2004 | ♂♀ | **NMNH**; CMNH; ZMUA | DOM |
| *H*. (*F*.) *shaamunensu* Dumas & Nessimian, 2019 | ♂ | **DZRJ** | BRA |
| *H*. (*F*.) *sigillata* Botosaneanu & Flint, 1991 | ♂♀ | **NMNH; ZMUA** | CUB |
| *H*. (*F*.) *singulare* Botosaneanu & Flint, 1991 | ♂ | **NMNH**; USNM; ZMUA | PRI |
| *H*. (*F*.) *sinuata* Denning & Blickle, 1979 | ♂ | **UCD**; NMNH | MEX; USA |
| *H*. (*F*.) *succincta* Johanson & Holzenthal, 2004 | ♂ | **NMNH**; UFBA | BRA; VEN |
| *H*. (*F*.) *sucrensis* Johanson & Holzenthal, 2004 | ♂ | **UMSP** | VEN |
| *H*. (*F*.) *tachira* Johanson & Holzenthal, 2004 | ♂ | **UMSP** | VEN |
| *H*. (*F*.) *tapadas* Denning, 1966 | ♂ | **CAS**; UFBA | BRA; VEN |
| *H*. (*F*.) *temora* Denning & Blickle, 1979 | ♂ | **UCD** | MEX |
| *H*. (*F*.) *thelidomus* Hagen, 1864 | C | - | VEN |
| *H*. (*F*.) *timbira* Silva, Santos & Nessimian, 2014 | ♂♀* | **DZRJ; MNRJ; INPA** | BRA |
| *H*. (*F*.) *truncata* Ross, 1956 | ♂ | **INHS;** UMSP; USNM | CRI; MEX; PAN |
| *H*. (*F*.) *turbida* Navás, 1923 | all | **MZBS**; NMNH; IBN; USNM | ARG |
| *H*. (*F*.) *tuxtlensis* Bueno-Soria, 1983 | ♂♀* | **IBUNAM**; UCD; USNM | GTM; MEX; PAN |
| *H*. (*F*.) *umbonata* Hagen, 1864 | all | **USNM**; MCZ; AMNH; USNM | JAM |
| *H*. (*F*.) *valligera* Flint, 1983 | ♂♀* | **NMNH**; USNM | ARG; BRA |
| *H*. (*F*.) *venezuelensis* Johanson & Holzenthal, 2004 | ♂ | **UMSP** | VEN |
| *H*. (*F*.) *vergelana* Ross, 1956 | all | **INHS**; NMNH; NRM; UCD; UFPE; UMSP; USNM | BLZ; BRA; COL; CRI; ECU; GTM; GUY; MEX; NIC; PAN; PER; SUR; TRI; VEM |
| *H*. (*F*.) *villegasi* Denning & Blickle, 1979 | ♂ | **UCD** | MEX |
| † *H*. (*F*.) *voigti* Johanson & Wichard, 1996 | ♂ | **Collection Wichard** | DOM |
| *H*. (*F*.) *woldai* Johanson, 2003 | ♂ | **NMNH**; UCD | PAN |
| *H*. (*F*.) *woytkowskii* Ross, 1956 | ♂♀* | **INHS**; NRM; UMSP | PAN; PER; VEN |

**Table 2.** Summary of the estimation of species richness in the new world based on incidence data with the CHAO2, second order jackknife (JACK2) estimators.

|  | **Chao2** | **Jacknife2** | **Average** | **Estimation of unknown species** |  |
| --- | --- | --- | --- | --- | --- |
| *Helicopsyche* (*Cochliopsyche*) | | | | | |
| Species estimates | 36 | 34 | 35 | 18 |  |
| error | 16 | 4 | 10 |  |  |
| *Helicopsyche* (*Feropsyche*) | | | | |  |
| Species estimates | 553 | 310 | 431 | 302 |  |
| error | 152 | 33 | 92 |  |  |
| New World *Helicopsyche* | | | | |  |
| Species estimates | 571 | 343 | 457 | 311 |  |
| error | 142 | 35 | 88 |  |  |

**Table 3.** Environmental variables used for species modelling, using 80% correlation cut-off.

| **Variables group** | **Code** | **Variables** |
| --- | --- | --- |
| Bioclimatic | Bio_7 | Temperature Annual Range (Annual Precipitation- Min Temperature of Coldest Month) |
|  | Bio_2 | Annual Precipitation |
| Elevation | Elev | Elevation |
| Precipitation | Prec_01 and 08 | Precipitation (mm) |
| Solar radiation | Srad_03, 04 and 07 | Solar radiation (kj m-2 day-1) |
| Wind speed | Wind_01 | Wind speed (m s-1) |

**Fig. Legends**

**Fig. 1.** Distribution and environmental suitability map for the New World Helicopsychidae species. A. Environmental suitability map (weighted average) of *Helicopsyche* (*Cochliopsyche*); B. Distribution records of *Helicopsyche* (*Cochliopsyche*) species; C. Map of high environmental suitability (>0.7) of *Helicopsyche* (*Cochliopsyche*); D. Environmental suitability map (weighted average) of *Helicopsyche* (*Feropsyche*); E. Distribution records of *Helicopsyche* (*Feropsyche*) species; F. Map of high environmental suitability (>0.7) of *Helicopsyche* (*Feropsyche*); G. Distribution records of New World Helicopsychidae species; H. Map of high environmental suitability (>0.7) of New World Helicopsychidae.

**Fig. 2.** Distribution range and species new species history of New World Helicopsychidae. A. Number of records and distribution range of *Helicopsyche* (*Cochliopsyche*) species. B. Number of records and distribution range of *Helicopsyche* (*Feropsyche*) species. C. History of new and number of *Helicopsyche* (*Cochliopsyche*) species over time. D. History of new and number of *Helicopsyche* (*Feropsyche*) species over time. E. Heatmap of distribution for *Helicopsyche* (*Cochliopsyche*). F. Heatmap of distribution for *Helicopsyche* (*Feropsyche*).

**Fig. 3.** Boxplot of biogeoclimatic variables of New World *Helicopsyche* species. A. Elevation range of *Helicopsyche* (*Cochliopsyche*) species; B. Elevation range of *Helicopsyche* (*Feropsyche*) species; C. Freshwater Stahler order range of *Helicopsyche* (*Cochliopsyche*) species; D. Freshwater Stahler order range of *Helicopsyche* (*Feropsyche*) species.

**Fig. 4.** Boxplot of biogeoclimatic variables of New World *Helicopsyche* species. A. Annual precipitation range of *Helicopsyche* (*Cochliopsyche*) species; B. Annual precipitation range of *Helicopsyche* (*Feropsyche*) species; C. Temperature range of *Helicopsyche* (*Cochliopsyche*) species; D. Temperature range of *Helicopsyche* (*Feropsyche*) species.

**Supporting information**

**S1 Table.** Summary of georeferenced distribution data for all New World Helicopsychidae species.

**S2 Table.** Summary of distribution in Freshwater ecoregion (*sensu* Abell et al. 2015) and limits data for biogeoclimatic variables of all New World Helicopsychidae species.

**S3 Table.** Pearson correlation between environmental variables.

**S4 Fig.** Boxplot with the result of Pearson correlation between environmental variables.

**S5 Fig.** Boxplot with result of evaluation of distribution modelling algorithms using the area under curve (AUC) method.
