# Supplementary material for: Addressing biodiversity knowledge shortfalls in New World Helicopsychidae (Insecta, Trichoptera): Potential distribution, environmental gradients, and identification of conservation and research priority areas
Source: PLoS One. 2025 Jul 28;20(7):e0327580. doi: 10.1371/journal.pone.0327580 (PMC12303351; doi:10.1371/journal.pone.0327580)
Supplement: Attachment — Submitted filename: Reponses to Reviewers.docx [file pone.0327580.s007.docx]

Dear,

Dr. Emily Chenette

Chief Editor of *PLOS One*

We would like to inform you that the manuscript was reviewed by PLOS One and received a response on 19/12/2023. However, due to a lack of funding at our institution, we were unable to proceed with the review process and decided to withdraw the manuscript. At that time, all requested corrections were made. During this period, additional data was included, and the manuscript went through several rounds of revisions by both the authors and reviewers from another free periodic, for which it was considered but ultimately rejected due to the page count not meeting their guidelines.

Therefore, the current manuscript is not identical to the one originally submitted, making it difficult to provide a point-by-point comparison of the revisions. However, we assure you that all requested modifications and improvements, as well as additional corrections, have been implemented. We decided to resubmit the manuscript now due to a financial support grant for publication, which will allow us to cover the required fees for publication in PLOS One, our first choice of journal due to the scope of the work and the quality of the publication.

Below, we have compiled the main comments from the reviewers and our responses to them.

We are happy to provide further details if needed and hope that our manuscript meets the expectations of the journal.

This manuscript does not violate any copyright or other personal proprietor of any person or entity, and it contains no abusive, defamatory, obscene, or fraudulent statements, nor any other statements that are unlawful in any way.

Thank you very much for your attention, and please let me know if I should provide any further information.

With best regards,

Rafael Pereira

1. Technical Soundness and Data Foundation

Reviewer #1:

The manuscript presents an interesting topic, but the biogeography section is not well explored. The conclusions could be more robust if the phylogenetic relationships among species were discussed more thoroughly. Additionally, the definition of bioregionalization and the choice of some analyses need further clarification.

Author's Response:

The biogeography section has been further explored in both the results and discussion, and the text has been improved to clarify patterns and relationships between species. The bioregionalization section has been removed, as we believe it will be better addressed in a multi-taxonomic perspective.

Reviewer #2:

The study is good, but there are limitations related to the use of a single genus, and bias in the resulting bioregionalization, which are not clearly discussed.

Author's Response:

The bioregionalization section has been removed, as we believe it will be more appropriately addressed from a multi-taxonomic perspective. However, accepting the suggestion, data from another Neotropical subgenus of Helicopsychidae have been incorporated.

2. Statistical Analysis

Reviewer #1:

The analyses are performed, but important details, such as the choice of "terrestrial ecoregions" to estimate unobserved species, need better explanation. Also, there is a lack of discussion on collection bias in the analyses.

Author's Response:

We accepted the suggestion and used the freshwater ecoregions from Abell et al. to estimate richness, as it seems more consistent for organisms that spend most of their life in freshwater environments.

Reviewer #2:

There are no specific suggestions, but some methodological issues need to be addressed more clearly.

Author's Response:

The suggestions were incorporated, and the Methods section was revised to make it clearer and more direct, allowing for reproducibility of the work.

3. Data Availability

Both reviewers confirm that the underlying data has been properly made available with no apparent restrictions.

4. Clarity and Quality of Writing

Both reviewers consider the manuscript well-written, but some parts could be revised to improve clarity, especially in the introduction and methods description.

Author's Response:

Suggestions for improving the writing in certain sections and correcting sentences and paragraphs that were difficult to understand were incorporated. A thorough review of the introduction was conducted, adding recent works, and the methods section was enhanced.

5. General Comments

Reviewer #1:

The main goal of the manuscript is not entirely clear, especially in relation to biogeography. It is suggested that bioregionalization be better discussed in terms of conservation and its specific characteristics. Furthermore, the role of phylogeny in the conclusions is not discussed, despite being relevant.

Author's Response:

The biogeography section has been more thoroughly explored in the results, discussion, and conclusion, and the text has been improved for clarity regarding patterns and relationships among species. The lack of comprehensive and current phylogenetic inferences, as well as the absence of consensus among the hypotheses presented so far, made discussions in this area difficult, but we believe the text has been enhanced from the previous version.

Reviewer #2:

The use of a single genus may limit the scope of the study. It is important to consider additional environmental variables, such as elevation, which affect species distribution. The concept of "pseudoabsence" needs to be better justified, and the analyses need to be clearer, especially concerning the formation of bioregionalization.

Author's Response:

The bioregionalization section has been removed, as we believe it will be better addressed from a multi-taxonomic perspective. However, accepting the suggestion, data from another Neotropical subgenus of Helicopsychidae [*H*. (*Cochliopsyche*)] have been incorporated. In the distribution modeling methods section, concepts have been more clearly justified, and the text on data analyses has been modified for better clarity.
